# How and Why to Replace the 14-Day Rule

**DOI:** 10.1007/s40778-018-0135-7

**Published:** 2018-07-16

**Authors:** Sarah Chan

**Affiliations:** 1 Usher Institute for Population Health Sciences and Informatics, Old Medical School, Teviot Place, University of Edinburgh, Edinburgh, EH8 9AG UK; 2 Centre for Biomedicine, Self and Society, University of Edinburgh, 23 Buccleuch Place, Edinburgh, EH8 9LN UK

**Keywords:** Embryo research, Ethics, 14-day rule, Stem cells

## Abstract

**Purpose of Review:**

The ‘14-day rule’, which limits research on human embryos to the first 14 days after fertilisation, has long been a pillar of regulation in this contested area. Recently, advances in developmental biology have led to calls to rethink the rule and its application. In this paper, I address the question of whether the 14-day rule should be replaced and, if so, how.

**Recent Findings:**

The two lines of research that have prompted this question are new techniques enabling culture of embryos at least up to 14 days and patterning experiments with pluripotent cells suggesting that they might form embryo-like structures. I consider each of these in relation to the foundations and function of the rule to examine whether they warrant change.

**Summary:**

I argue that the 14-day rule for embryo research should be open to change, but that this possibility must be addressed through early and thorough discussion involving a wide range of publics and other stakeholders.

## Introduction

The ‘14-day rule’ is a staple of human embryo research governance. Though implemented in various ways in different countries, its essence is the same wherever the limit applies: it stipulates that human embryos, for whatever purpose, should not be grown in vitro for longer than 14 days after the point of fertilisation.

The adoption of the 14-day rule in public policy is generally attributed to two major points of origin: in the USA, the 1979 report of the Ethics Advisory Board to the Department of Health, Education and Welfare (HEW) on embryo research [1] and, in the UK, the report of the Warnock Committee of Inquiry into Human Fertilisation and Embryology [2]. From these foundations, the rule has acquired widespread influence elsewhere: almost every country in which embryo research is specifically permitted by regulation, soft or hard, employs a version of the 14-day rule.

For this reason, recent suggestions that the rule may be due for revision have caused ripples in the science, ethics and policy community worldwide.

## Ethical and Policy Significance

In order to understand the significance of the 14-day rule and therefore why and whether replacement might be warranted, it is useful to explore the foundations of the principle: why 14 days?

The central point of contention in embryo research has, from the outset, been the moral status of the human embryo and whether it is permissible to use and destroy embryos in pursuit of research [3–5]. At one extreme, some maintain that the embryo, purely in virtue of being a human life (or, a related but distinct argument, having the potential to develop into a human being [6–9]), has moral status equal to any other human being [10, 11], and harming or destroying it in the course of research would therefore be ‘in-principle’ wrong [12]. At the other extreme, some hold that moral status is based on features and capacities none of which the embryo possesses [13–17], that the embryo is ‘simply a collection of cells’ ([2] at 11.15) and that embryos therefore deserve no special protection.

Between these two poles lie a range of intermediate opinions with varied justifications. These include gradualist views, according to which the embryo acquires moral status during the process of development, starting from no or very little moral status and eventually acquiring the full moral status usually associated with human persons [18]. This seems a plausible notion and accords with most people’s intuitions, but leaves us with the difficulty of determining at what point the threshold of moral status is reached such that research would no longer be permissible. Another intermediate position, based on different reasoning, is that the embryo may not have intrinsic moral value or moral status *sui generis*, but has some value or deserves respect for other reasons, for example its instrumental value to others or its symbolic value as the beginning of human life [19, 20].

The Warnock Committee devoted serious attention to the problem of how the human embryo should be treated, while explicitly demurring to address the question of *moral* status and whether the embryo should be considered a ‘person’ in the moral sense. Like the advisory group responsible for the HEW report, they favoured allowing some research to proceed, but recognised that the sensitive and highly controverted nature of the issue required that embryos be granted some sort of ‘special status’ and that ‘stringent controls and monitoring’ be imposed in order for embryo research to be permitted (reviewed in [21•]).

The 14-day solution eventually decided upon had some moral foundations. In selecting 14 days as the limit, the Warnock Committee referred to sentience (the ability to feel pain) and individuation (the emergence of a definite single human individual) as relevant factors in their determination ([2] Chapter 11, [22]). In noting that neural development begins at around 17 days and ‘subtracting a few days’ from this to ensure ‘that there would be no possibility of the embryo feeling pain’ ([2] 11.20, [21•]), and going on to describe the formation of the primitive streak after 14 days as ‘the beginning of individual development’ ([2] 11.22), it thus tied biological and moral considerations neatly together into a countable and defined criterion for the acceptability of embryo research.

Reading a definite moral position on the status of the human embryo into the 14-day policy, however, is problematic. The arguments presented by Warnock in favour of using 14 days as the cut-off point provided some assurance that before this point, there did not exist a single identifiable being about whom we should be concerned. They did not imply that after this point there *would* exist such a being, that 14 days represented some sort of ‘bright line’ between the embryo having no moral status and suddenly acquiring it. In fact, the 14-day limit did not reflect a firm position on the embryo’s *moral status* at all. Instead, both in the USA and the UK, it brokered a sort of policy compromise [23, 24•, 25•] between groups with radically different interests and views on the moral legitimacy of embryo research.

In the UK, the work of the Warnock Committee led to the Human Fertilisation and Embryology Act of 1990, establishing a regulatory system that has been in place since. In the process, it solidified the terms of the relationship between potentially controversial science and diverse publics with varying moral views and interests, and the role of policy-oriented bioethics in negotiating this [26]. As debates over human embryonic stem cell research and, later, new forms of embryo research and reproductive technologies unfolded in subsequent decades, the foundations laid for this mode of addressing ethical issues through science policy enabled new issues to be resolved relatively smoothly [27].

Throughout these debates, the 14-day rule remained a central element of regulation. More than exactly *when* the cut-off point was set, the existence of *some* limit, determined via moral deliberation and bearing political legitimacy, together with regulatory mechanisms to ensure that the rules were kept and sufficiently serious sanctions (in the form of criminal penalties [28]) to indicate the gravity of the issue, was a key factor in successful regulation [29]. Although the regulation of embryo research in the USA developed along different lines, via guidelines rather than hard law and with the added complication of rules about federal funding, the 14-day limit likewise remained a key feature.

Thus, though not a moral principle in itself, the 14-day rule has nevertheless performed, and continues to perform, crucial ethical work via its function in embryo research policy.

## Why Change?

The 14-day rule, whether implemented by legislation or guidelines, has functioned successfully for decades: why, then, might we think to change it? Suggestions that change is needed have arisen recently for two reasons, both related to recent scientific developments that we might say render the rule simultaneously too restrictive and not restrictive enough.

When the 14-day limit was set, it was largely theoretical: human embryos could not then be grown in culture for more than a few days. Now, however, culture techniques and understanding of early embryonic development have advanced to a point where it might well be possible to continue maintaining embryos in vitro beyond 14 days. In 2016, two groups of scientists reported that they had succeeded in growing embryos past the stage when implantation would usually occur in vivo (approximately 7 days), which had previously been a technical limitation [30, 31]. Using new protocols for in vitro culture, they were able to continue growing the embryos in order to study the post-implantation stage of development. Both groups terminated the experiments at 13 or 14 days, citing the internationally recognised ethical guideline of the 14-day rule as the reason.

It seems, therefore, that exceeding the 14-day limit is probably now technically possible. Of course, ‘can’ is not ‘should’: the mere fact that something is possible does not mean we ought to do it, or that it is morally permissible. (Cavaliere, in her analysis of the 14-day rule, calls this the argument from ‘technical feasibility’ and rejects it on this same basis [25•].) It is becoming apparent, however, as experiments approach the 14-day limit, that useful scientific knowledge may lie beyond this boundary. For example, the appearance of the primitive streak marks the start of gastrulation, a key developmental event; culturing embryos through this phase and beyond would allow this process to be studied. Again, this does not automatically render such research morally permissible. If there are benefits to be gained, however, these weigh in favour of allowing research, providing a reason at least to reconsider whether a revision of the current rule might be justified.

The second development is the discovery that human pluripotent cells, when grown under certain culture conditions, can self-organise in ways similar to the human embryo [32]. While these ‘gastruloids’ are still very different from actual embryos, the prospect of groups of cells that can recapitulate aspects of embryonic development without being embryos in the usual sense of developing from a single zygote, whether produced by fertilisation or nuclear transfer, raises ethical and regulatory questions [33, 34•, 35•]. Particularly in terms of the 14-day rule and its application, it is unclear whether such objects would fall within the definition of embryos and be subject to regulation as such. Moreover, even if they were, there is no ‘day zero’ from which to begin counting. Is a new approach to regulation required for these experiments?

The 2016 ISSCR guidelines attempt to account for this possibility by extending the application of the limit to ‘organized embryo-like cellular structure[s] with human organismal potential’ ([36] 2.1.3.3(b)). There is, however, a problem in determining ‘human organismal potential’: how will we know whether these structures possess such potential, without allowing them to develop past the point at which this capacity (or lack of it) would be evident? This poses what we might call the ‘14-day paradox’: it is precisely those processes that are important to normal embryonic development and that need to be studied in order to understand development that the 14-day limit is designed to avoid.

In considering this issue, Aach and colleagues refer to these groups of cells as ‘synthetic human entities with embryo-like features’, or ‘SHEEFs’ [35•]. (Even to call something an ‘entity’ is problematic, in that it implies some kind of individuality or subjecthood; by comparison, we do not generally refer to cell lines, ES colonies or organoids as entities.) They raise the possibility that SHEEFs might develop ‘morally concerning properties’ without passing through the stages of ‘canonical embryogenesis’ that were used in determining the limit for embryo research and suggest that research limits for SHEEFs should instead be based on ‘moral status signifying features’. As they themselves note, however, the relationship between moral status and developmental features is not straightforward; the relationship between the moral status of embryos and how we should regulate them is even less so.

## What Is an Embryo?

The uncertain nature and status of what is created by ‘gastruloid’ or ‘SHEEF’ research highlights a conceptual difficulty. To understand how they should be treated, in view of the ‘kind of thing’ that they are, requires us to answer the question: just what ‘kind of thing’ are they?

The problem this raises, of ‘what is an embryo’—biologically, morally and ontologically—recalls discussions that took place over somatic cell nuclear transfer [37, 38] and embryo splitting [39], as well as over human embryonic stem cell (hESC) research and the associated concerns regarding various levels of potency that embryos, blastocysts and isolated cells might possess. Common to these discussions was that they sought to determine what makes an embryo *biologically*, in order to be able to demarcate what might be special about it *morally*.

This preoccupation with attempting to map the moral nature of the embryo onto its biological nature led, in one case, to a proposal to create embryos by a process of ‘altered nuclear transfer’ [40–42], such that they would lack full developmental potential and hence (in the eyes of those for whom the embryo’s potential was a reason to accord it moral status) be legitimate subjects for experimentation. This suggestion, though never demonstrated to be practicable, much less implemented, represented probably the peak example of attempts to render embryos somehow ‘un-embryo’ as a way of solving the moral problems associated with their use. More recently, the conflation of biology and morality has occurred in relation to mitochondrial replacement therapy (MRT). Questions about the identity of an embryo created by MRT have played a significant role in both bioethical and public discourse; in disrupting the biological basis of the embryo, identity itself, its relationship to biology and its ethical significance become problematized [43–46].

At least in relation to the embryo problem, turning to biology to solve moral problems has not been wildly successful so far. In searching for biological answers to moral questions such as what defines identity or what sorts of things should be accorded moral status, we are finding that biology is inadequate to define morality. Indeed, as we drill down further into the biology via gastruloid and later-stage embryo research, the moral problems become more, rather than less, difficult.

## Moral Concern and Misdirection

In fact, in deciding whether to replace the 14-day limit, we ought not to be looking primarily to the properties of SHEEFs or embryos themselves. Consider this: what are the possible ‘morally concerning properties’ that post-14-day embryos or SHEEFs might develop? Obviously, sentience—the capacity to feel pain—is one, but merely because a being can feel pain does not mean it is wrong to conduct research on it or that it is harmed by research, only that it would be wrong to cause that being to suffer. Animal welfare and non-human animal research regulations account for these concerns, yet we do not think them appropriate or sufficient to govern research on human embryos.

The mistake here is to concentrate on the properties or capacities gradually acquired by the developing embryo, rather than other factors that make, or might make, a human embryo of moral concern. A focus on development of ‘morally concerning properties’ is misdirected: the original moral concern over embryos and how we ought to treat them, which still persists, was not and is not primarily based on their possession of these properties. Attempting to adopt this approach to determine policy is to roll back to a stalemate of diametrically opposed, irreconcilable views on the human embryo: as a ball of cells possessing none of the capacities usually associated with having moral interests, versus an entity due the same respect as a human person and on which it would be unacceptable to experiment, because of its property of humanness, potentiality to become a human, or symbolic value as the start of human life. Such positions are irreconcilable precisely because they are based on different accounts of the properties that create moral value.

For this reason, the treatment of the human embryo under UK law is not based on its *moral status* as such but what the Warnock report referred to as ‘special status’, related as much to the social positioning of the embryo as its intrinsic properties. Recognising this status and respect for it was compatible with setting the limit in the first place and allowing research to proceed; it could also be compatible with moving it, but regulating embryos on the basis of their properties is not the solution.

## Crossing the Limit?

In considering the possibility of extending the 14-day limit, it is perhaps useful to reflect on what crossing this boundary might entail. According to the current regulatory position, before this point, embryos are something that it is legitimate to experiment upon, but afterwards? What are we afraid of, in contemplating the possibility of crossing this limit: what actually happens at 14 days?

Certainly, the post-14-day embryo does not suddenly become something inviolable or deserving of moral protection. In all probability, it would most likely fail to develop much further; we as yet lack sufficient understanding of the complex signals provided by the intrauterine environment and the processes by which they interact with the developing embryo to be able to simulate them well enough to allow continued development. Indeed, understanding these signals and why they sometimes fail, leading to early pregnancy loss, is one of the possible benefits cited in favour of allowing this research.

But imagine if, by some chance, the embryo were able to survive and continue some form of development in vitro. Either, were development able to continue more or less normally, it would at some point presumably become something deserving of moral protection in itself, a being with moral rights who should not be experimented on; or it would not, in which case, it would matter only instrumentally (that is, in terms of the interests of others) what we do with it. In either case, we should treat it accordingly; so far, so good.

Our primary concern about such a possibility, then, is not with what the moral status or moral interests of the being a SHEEF or in vitro embryo might possibly become. Instead, this possibility enters the realm of human-animal chimeras, cloned embryos and germline genetically modified humans: ‘uncanny beings’ that produce a sense of deep moral unease (or ‘inexorable moral confusion’ [47]), but not because their existence would necessarily be a catastrophic moral wrong to them or to others.

Justifying this feeling of unease and coming up with reasons why this should not be permitted often relies on vague statements about human dignity [10, 48, 49] or moral confusion. Critics of such appeals to dignity, however, contend that they have thus far not produced a robust account of what would be wrong about such cases and how [50–53]. Perhaps we just do not know; the creation of such beings, and the scope, scale and nature of the wrongs or rights it might entail, remains a sort of moral *terra incognita*.

Does that mean, then, it would be better not to allow things to get to that point—that we should not cross the boundary for fear of the moral unknown? It might at least be wise not to venture into the wilderness without a map. Two points, though, follow from this.

First, we would still be a long way off the point of growing walking, talking, viable human beings in vitro even if the limit were revised upwards to, say, 28 days. A modest revision to the limit ought not to take us into ‘uncanny’ territory. Second, however, we should start ‘map-making’—developing new ethical approaches to better delineate and analyse the issues that these novel beings might raise. It is not out of the question that, in the same way the first baby born via mitochondrial replacement transfer was announced to the world at large after the fact [54, 55], someone may one day soon announce the first germline genome-edited baby; the first baby born by ectogenesis or from a ‘synthetic embryo’ is a more far-fetched possibility, but one for which we ought to be ethically prepared.

## Slippery Slopes

A final issue that must be addressed in considering whether to rethink the 14-day rule is the problem of the slippery slope.

Slippery slope concerns were prominent in the early embryo research debates and have continued to surface since. Critics feared that allowing research even on early embryos would be a first step down a path which, once on, we would be unable to stop following; that even if such research were ethically permissible in itself, it would inevitably lead to pushing the limit further and further, past some eventual point which would be unacceptable.

Proposals to change the 14-day rule might be particularly vulnerable to such accusations, since they may be viewed as evidence of the slope’s slipperiness. Further, because the limit has so often been cited as a safety net in response to slippery slope concerns, removing or changing the limit might undermine public confidence in the ability of regulation to police and maintain appropriate boundaries. For these reasons, it would be worth attempting a fuller analysis of the ’slippery futures’ that were foreseen, whether they have come to pass, and how we ought therefore to respond to slippery slope arguments about embryo research in the present. Space does not permit such an endeavour here; we can, however, make some general observations.

In the first place, simply because the limit can be moved does not imply that the slope is inherently slippery, or even that it is a slope at all. While it stands in regulation, the limit acts as a fence from behind which we can safely assess the territory that lies on the other side, before deciding whether or not to move the fence and cross the line on which it currently sits.

We might also argue that, in policy-making at least, some slopes ought to be slippery; policies made in controversial areas should be open to change, recognising the possibility of evolving norms and moral thinking. Laws granting voting rights to women, removal of laws against miscegenation, introduction of anti-discrimination legislation and legalisation of same-sex marriage are all examples of what some might call slippery slopes but most would probably count as moral progress. Unless we think we have already reached the pinnacle of ethical and scientific knowledge, that no further development of moral understanding is possible nor will science present us with any challenges that our existing moral framework cannot easily solve, then policy should be open to change—indeed, will need to change—to address the novel possibilities that new scientific knowledge opens up. Far from proof of a slippery slope, a willingness to revisit rules is, we might argue, a necessary part of ethical evidence-based policy.

## Conclusions: How to Change?

Asking ‘how to change’ the 14-day rule implies that a change is necessary. Perhaps, then, a better question would be how to revisit the rule and assess whether change is appropriate and, if so, what form it should take.

For the purposes which the rule serves, it is probably better to stick with an objective, time-based limit rather than determination of features whose emergence, and the assessment thereof, may vary. As Baroness Warnock commented, ‘anyone can count to 14’; it is less certain that ‘anyone’ can confidently say when a developing embryo might start to feel pain. Even a clear biological feature such as the primitive streak, obvious to embryologists and developmental biologists, is arcane and far-removed from the everyday experience of anyone who does not work with embryos in the laboratory, and thus a tenuous foundation for public confidence in regulation. Basing the legitimacy of embryo research on a subjective determination of the emergence of ‘morally significant features’, resting primarily in the hands of the experimenters, would not satisfy publics’ need to be assured of oversight for this sensitive area of science, nor provide the same degree of security to scientists working with embryos. Besides this, there is also the problem that setting a limit with reference to the emergence of some feature *before* which development should be halted presents a practical difficulty: how is one to know where the limit is before one has already gone past it?

A limit based on time from initiation of normal embryonic development does still leave the problem of SHEEFs and how they ought to be regulated. However, the SHEEF problem may be something of a red herring in the debate over extending the 14-day rule. SHEEFs, whatever ‘kind of thing’ they are, should not at present be regulated as if they were the same ‘kind of thing’ as embryos: ontologically and thus ethically, they are not. It may seem inconsistent to say that two objects with similar functional properties and potential should be treated differently because of their origin or destiny. We should remember, however, that the prescribed ways in which embryos or SHEEFs ought to be treated are not based on their moral status per se but on their positioning with respect to society, and in this regard, SHEEFs and embryos are different.

Further, given the significance of the 14-day rule as a boundary object [56, 57] that has enabled cooperation over embryo research in the face of continued moral controversy, renegotiating the terms of the process should be handled with caution. If done carelessly, it might threaten to disrupt the so-far reasonably harmonious relationships between science, society, ethics and policy. This does not, though, mean the limit cannot be revisited at all; lines can be redrawn while still respecting the process that originally drew them. As Cavaliere argues, ‘the approval of MRTs shows that longstanding limits such as the ban on germline modification can be redefined’ [25•]; the challenge then is how to approach the revision. Engagement, consultation and wide-ranging discourse will be essential to maintain these relationships and ensure that the outcome retains legitimacy and effectiveness as a form of governance.

There is one important caveat: we should avoid the assumption that the role of publics in this process is necessarily to object and obstruct. The construction of an adversarial dynamic between science and publics has negative effects, and our job as ethicists and policy-makers is more than just to ‘prevent public backlash’ [24•]. Certainly, there will be voices opposed to any extension of the 14-day limit, but there will also be those, not just scientists, who support it; there will be fears and concerns, but also hopes attached to this new area of research. In fact, a poll conducted in the UK in 2017, in the wake of early suggestions to change the rule, showed almost half of respondents in favour of extending the limit [25•]. Regardless of the numbers, however, avoiding debate in an attempt to silence dissenting voices would not be congruent with a democratic approach to the governance of science [58].

We should also consider carefully the transnational dimensions of regulatory change and how this ought to be managed. Will the first country to permit embryo research beyond 14 days become a destination for scientific tourism? What pressures might this create for change in other countries, and how will this affect the global dynamic of science? Again, similar concerns were raised around hESC research [59, 60]. One thing that has become increasingly clear, as the regulatory debate over ethically challenging technologies has progressed through various iterations (and with varied success), from cloning to gene editing, is that a coordinated international approach is essential: if we are to contemplate replacing the 14-day limit, we must do so together.
